# Feasibility of Using Pseudo-Continuous Arterial Spin Labeling Perfusion in a Geriatric Population at 1.5 Tesla

**DOI:** 10.1371/journal.pone.0144743

**Published:** 2015-12-14

**Authors:** Sigurdur Sigurdsson, Lars Forsberg, Thor Aspelund, Rob J. van der Geest, Mark A. van Buchem, Lenore J. Launer, Vilmundur Gudnason, Matthias J. van Osch

**Affiliations:** 1 The Icelandic Heart Association, Kopavogur, Iceland; 2 The University of Iceland, Reykjavik, Iceland; 3 Department of Radiology, Leiden University Medical Center, Leiden, The Netherlands; 4 Laboratory of Epidemiology, Demography, and Biometry, National Institute on Aging, National Institutes of Health, Bethesda, MD, United States of America; University Medical Center (UMC) Utrecht, NETHERLANDS

## Abstract

**Objectives:**

To evaluate the feasibility of using pseudo-continuous arterial spin labeling (pCASL) perfusion in a geriatric population at 1.5-Tesla.

**Materials and Methods:**

In 17 participants (mean age 78.8±1.63 years) we assessed; 1) inter-session repeatability and reliability of resting state perfusion in 27 brain regions; 2) brain activation using finger-tapping as a means to evaluate the ability to detect flow differences; 3) reliability by comparing cerebral blood flow (CBF) with pCASL to CBF with phase contrast (PC-MR).

**Results:**

The CBF (mean±standard deviation (SD)) for the whole brain grey matter (GM) was 40.6±8.4 and 41.4±8.7 ml/100g/min for the first and second scan respectively. The within-subject standard deviation (SDw), the repeatability index (RI) and intra-class correlation coefficient (ICC) across the 27 regions ranged from 1.1 to 7.9, 2.2 to 15.5 and 0.35 to 0.98 respectively. For whole brain GM the SDw, RI and ICC were 1.6, 3.2 and 0.96 respectively. The between-subject standard deviation (SD_B_) was larger than the SDw for all regions. Comparison of CBF at rest and activation on a voxel level showed significantly higher perfusion during finger tapping in the motor- and somatosensory regions. The mean CBF for whole brain GM was 40.6±8.4 ml/100g/min at rest and 42.6±8.6 ml/100g/min during activation. Finally the reliability of pCASL against the reference standard of PC-MR was high (ICC = 0.80). The mean CBF for whole brain measured with PC-MRI was 54.3±10.1 ml/100g/min and 38.3±7.8 ml/100g/min with pCASL.

**Conclusions:**

The results demonstrate moderate to high levels of repeatability and reliability for most brain regions, comparable to what has been reported for younger populations. The performance of pCASL at 1.5-Tesla shows that region-specific perfusion measurements with this technique are feasible in studies of a geriatric population.

## Introduction

Arterial spin labeling (ASL) can be used to visualize and quantify cerebral blood flow (CBF), an important physiological parameter used in the diagnosis and assessment of neurological disorders as well as for an examination of brain function [1]. Several studies have shown high reliability of ASL techniques [2–5]. Most of these studies have been limited to 3-Tesla field strength and whole brain cortical grey matter (GM). The advantage of increased field strength is higher signal-to-noise ratio (SNR) which results from a combination of higher intrinsic SNR and longer T1-relaxation time of both tissue as well as arterial blood [6]. The ASL signal decays with time constant T1 after labeling [6]. The reported T1-relaxation time of arterial blood at 1.5-Tesla is in the range of 1350 msec to 1530 msec [7, 8], comparable to or even shorter than the arterial transit time for the flow of blood from the labeled region to the imaging region. Arterial transit times in healthy GM can vary between 500 msec and 1500 msec but can be 2000 msec or longer in cerebrovascular disease and in deep white matter (WM). Ideally the post-labeling delay (PLD) should be just longer than the longest arterial transit time. However, due the rapid decay of the ASL signal with time constant T1, it is too costly in terms of SNR to always meet this requirement. This is less of a problem at 3-Tesla field strength where arterial blood T1-relaxation times are longer compared to 1.5-Tesla (approximately 1650 msec) [6, 9].

However, ASL techniques are emerging as a tool for clinical use and the availability of systems at field strength of 1.5-Tesla are more widely available. Further, most extant studies are limited to young subjects. There is a need for reliability studies in older persons where there are specific issues that need to be accounted for when aiming for reliable measures. Older persons are at high risk for neurodegenerative disorders, and more detailed assessment of localized brain regions is needed. Motion artifacts in MR images that degrade their quality and clinical utility may be more likely to occur in older compared to younger adults. Imaging with ASL of older persons is also challenging due to age related morphological alterations of vasculature such as increased vessel tortuosity and damage of arteriole walls [10] shown to alter transit times and dispersion of blood flow tracers [11] resulting in inaccuracies in perfusion measurements. Further, age related atherosclerotic vascular disease including steno-occlusive disease can result in longer arterial transit times of blood water [12].

The objective of this study was to evaluate the feasibility of using pseudo-continuous arterial spin labeling (pCASL) in very old individuals with a commercial 1.5-Tesla MRI system. To our knowledge, there is no other study in the current literature that has assessed pCASL reproducibility in localized brain regions in a geriatric population at 1.5-Tesla.We assessed the following; 1) inter-session repeatability and reliability of resting state perfusion in template driven brain regions including GM and WM with and without white matter hyperintensities (WMH); 2) task-driven brain activation in GM regions as a way to assess the ability of pCASL to detect flow changes; and 3) reliability of pCASL by comparing whole brain CBF values generated with pCASL to whole brain CBF values generated with phase contrast -MRI as a reference standard.

## Materials and Methods

### Subjects

The volunteers for this study were recruited from participants in the prospective population based Age Gene/Environment Susceptibility-Reykjavik Study [13]. Repeated pCASL scans were acquired in 17 healthy subjects (7 women and 10 men); age (mean±standard deviation (SD)) 78.8±1.63 years in June 2012. Imaging with pCASL was performed three times; during rest, and then in the same scanning session during activation by bilateral finger tapping. Following this session and a 15 minute pause, each subject was repositioned in the scanner and rescanned during rest. Study subjects were requested to stay awake with eyes closed when the scan was being acquired. The study was approved by the Icelandic National Bioethics Committee, which acts as the Institutional Review Board for the Icelandic Heart Association. Written informed consent was obtained from all participants.

### MR acquisition

**Fig 1:**
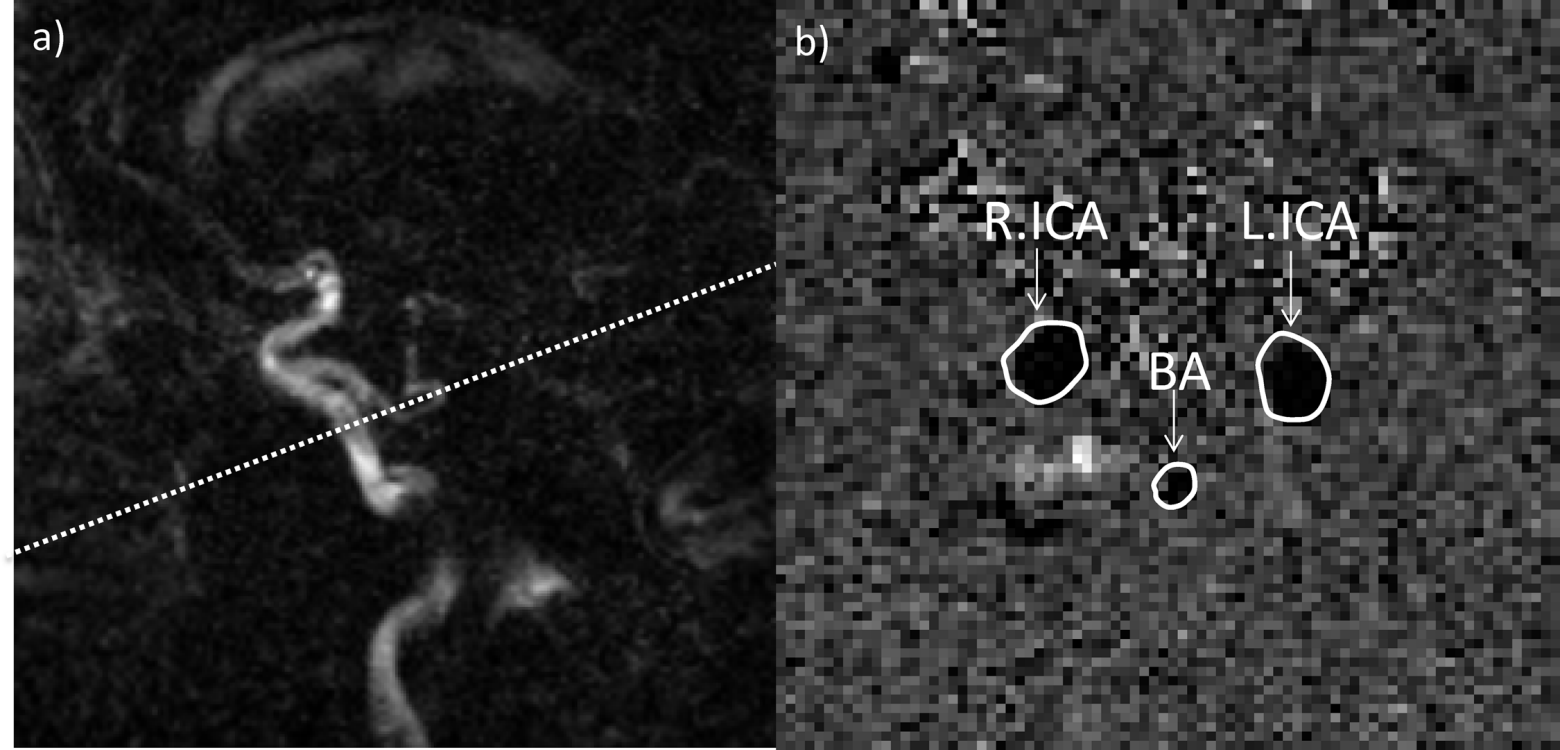
PC-MRI for quantification of total CBF. (a) A PC-MRI scan for measuring mean tCBF was prescribed on a PC-MRI sagittal localizer image perpendicular to the carotid arteries at the level of the mid basilar artery. (b) A representative phase image demonstrating the right and left internal carotid arteries (R.ICA and L.ICA) together with the basilar artery (BA).

All scans were acquired on a 1.5-Tesla Signa system (General Electrics (GE), Waukesha, WI). In the first session when subjects were at rest, imaging with pCASL was followed with phase contrast (PC)-MRI for quantification of total CBF (tCBF). Anatomical imaging was acquired for brain tissue segmentation. The pCASL imaging was performed with the product sequence from GE [14], which adheres almost completely to the recently internationally recommended implementation of ASL [6]; a 3D fast spin echo (FSE) using spiral acquisition and background suppression with a scan time of 5 minutes (TR ⁄TE = 4678 ⁄ 9.8 msec, labeling duration 1500 msec, post labeling delay 1525 msec, 512 sampling points on eight spirals; reconstructed matrix 128x128, three averages, slice thickness 4 mm, FOV 24 cm). A PC-MRI scan (TR ⁄TE = 20⁄6.2 msec; flip-angle, 9°; FOV, 22 cm; matrix, 256x256; slice thickness 5 mm; velocity encoding 100 cm/sec) for measuring mean tCBF was prescribed on a PC-MRI sagittal localizer image perpendicular to the carotid arteries at the level of the mid basilar artery (Fig 1). The anatomical image protocol has been described in detail elsewhere [15] and included T1-weighted 3D spoiled gradient echo, proton density/T2-weighted FSE and fluid attenuated inversion recovery (FLAIR) sequences.

### Image Processing

#### Quantification of perfusion

The quantitative CBF maps were generated using software (v16, M4) on the GE scanner console computer with a method proposed by Wang et al [6, 16]. In brief, the flow was calculated using the following equation:

$$f=\;\frac{\lambda }{2aT_{1b}\left(1-e^{-\frac{\tau }{T_{1b}}}\right)}\frac{\left(S_{ctrl}-S_{lbl}\right)\left(1-e^{-\frac{t_{sat}}{T_{1g}}}\right)}{S_{ref}}e\frac{w}{T_{1b}}$$ (1)

where *f* is the perfusion, S is the signal on the control- (S_ctrl)_, label- (S_lbl_) or reference image (S_ref_), λ is the blood brain partition coefficient (0.9 ml/g), α is the labeling efficiency (assumed to be 0.80), T_1b_ is the T1 of blood (assumed to be 1400 msec). The same T1 of blood was assumed for men and women [6]. The partial saturation of the reference image is corrected by using a T1_g_ of 1200 msec (assumed T1 of GM), t_sat_ is the saturation time (2000 msec), w is the post-labeling delay and τ is the labeling duration.

#### Brain tissue segmentation

**Fig 2:**
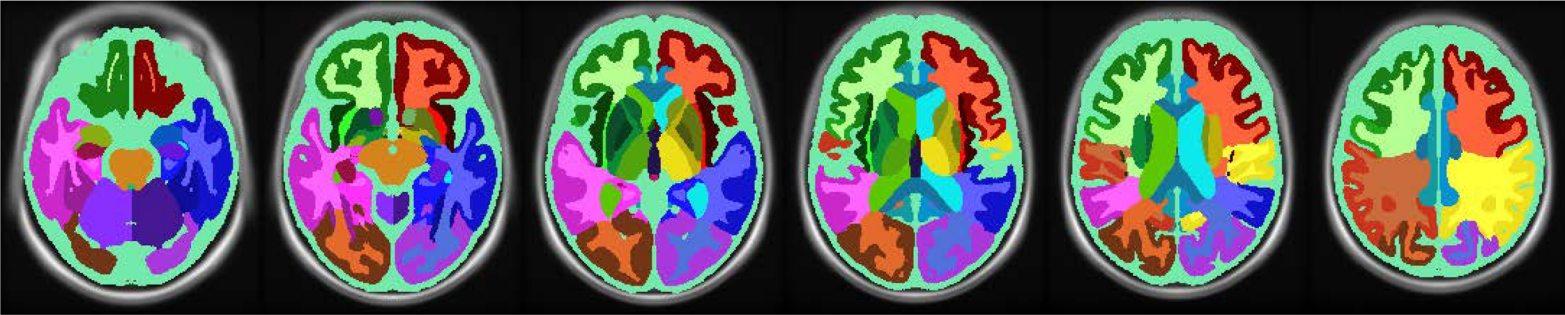
Anatomical atlas for regional segmentation. Regional segmentation was done by non-linearly warping an atlas of 56 regions to the T1-weighted image.

Whole brain tissue segmentation of GM, WM, WMH and cerebral-spinal-fluid (CSF) was done on intensity normalized and non-uniformity corrected anatomical images in MNI Talairach space, using a trained classifier [15]. Regional segmentation was done by non-linearly warping an atlas of 56 regions to the T1-weighted image (Fig 2). The average CBF values were calculated for 27 regions of interests (ROIs); whole brain, whole brain GM, whole brain WMH, normal appearing WM and total WM in addition to 22 other GM and WM regions excluding WMH. Right and left hemispheric regions were combined into one. Normal appearing WM was defined as white matter without WMH and total WM as white matter including WMH. Whole brain volume was defined as the sum of whole brain GM- and total WM volumes [15].

#### Brain activation

For assessing the ability of pCASL to detect flow differences, statistically significant differences in perfusion between brain activation and rest were determined voxel-wise within MNI-space. Testing for significance was performed by permutation testing using the FSL software randomize with 5000 permutations [17]. The permutations were enhanced with Threshold-Free Cluster Enhancement [18] resulting in a 1-P image, corrected for family-wise error.

#### Quantification of tCBF

The PC-MR images were processed using the software package FLOW (Division of Image Processing, Leiden University Medical Center, The Netherlands) [19]. The software quantified the average blood flow volume to the brain (in ml/min) after correcting for background velocity in stationary tissues. The average blood flow volume was subsequently divided by the whole brain volume to generate average whole brain CBF expressed in units of ml/100g/min assuming an average brain density of 1.05 g/ml [20].

### Statistical analysis

The repeatability and reliability of pCASL was assessed by comparing the CBF measurements from the two scans at rest in the 27 ROIs. Several statistical parameters were included in these assessments that individually or all together have commonly been used in previous studies addressing ASL perfusion repeatability including Bland-Altman plot demonstrating the agreement between the first and second scan at rest for whole brain CBF [2, 4, 21]. The CBF for all ROIs were tested for normal distribution. Subsequently, a paired t-test was used to assess statistically significant difference in mean CBF between the two scans. Secondly, the within-subject SD (SD_w_), a measure of measurement error was calculated as the square root of the residual mean square within subjects using one-way ANOVA [22]. The between-subject SD (SD_B_) was calculated in the same way as SD_W_ but from residual between subjects mean square. Third, the repeatability index (RI), defined as the 95% confidence limits for the difference between repeated measurements was calculated using [23]:

$$RI=\sqrt{2\;\text{x}\;1.96\;\text{x SDW}}$$ (2)

Fourth, the within-subject coefficient of variation (CoV) which quantifies the measurement error relative to the size of the measurements was calculated by dividing the SD_w_ with the overall mean of CBF for both scans [24]. Lastly, the intraclass correlation coefficient (ICC) was used to estimate the reliability of measurements. The ICC was estimated from a random effects model using PROC MIXED. An ICC close to 1.0 indicates high reliability, ICC values between 0.5 and 0.8 indicate a moderate reliability, whereas a value of 0.5 or lower indicates a randomness of results having limited use in distinguishing subjects [24].

For assessing the ability of pCASL to detect flow differences, statistically significant differences in perfusion between brain activation and rest were determined voxel-wise after correcting for multiple comparisons.

For the assessment of reliability of pCASL compared to PC-MRI, the ICC was calculated and a paired t-test used to assess difference between whole brain resting mean CBF values generated with the two techniques. Level of significance for all analyses was set to <0.05. All statistical analyses were performed with SAS/STAT®9.2 (SAS Institute Inc).

## Results

### pCASL—Repeatability and reliability

Study sample characteristics are summarized in Table 1. In addition to volumes for the 27 brain ROIs, Tables 2 and 3 include by region the mean CBF with SD_B_ for the two pCASL scans acquired at rest, and the measures of repeatability and reliability. There was no statistically significant difference between the two resting scans for any of the 27 ROIs except for the occipitotemporal gyrus (p = 0.04). The SD_B_ was larger than SD_w_ in all regions. There was a significant negative correlation between regional volume and SD_w_ (-0.83, <0.0001). Small ROIs had generally greater within-subject variability compared to large ROIs.

**Table 1:** Characteristics of the study group (n = 17) by sex.

Demographics	Overall, n = 17	Men, n = 10	Women, n = 7
Age, mean±SD	78.82±1.59	78.90±1.66	78.71±1.60
Range	76–82	77–82	76–80
ICV* (ml), mean±SD	1563.0±128.0	1626.1±119.2	1472.8±79.0
Whole brain fraction (%), mean±SD	71.2±3.3	70.2±3.3	72.7±2.8
GM fraction (%), mean±SD	43.8±3.1	42.6±3.3	45.5±1.9
Normal WM fraction (%), mean±SD	25.9±1.7	25.4±1.5	26.4±2.0
WMH fraction (%), mean±SD	1.6±1.7	2.1±1.8	0.8±0.5
Height (cm), mean±SD	170.8±9.5	177.3±6.0	161.7±4.6
Systolic BP (mmHg), mean±SD	139.6±18.9	143.4±22.4	134.3±12.2

ICV = Intra Cranial Volume, Brain fraction = brain parenchymal volume as a fraction of ICV, GM fraction = grey matter volume as a fraction of ICV, NWM fraction = normal white matter volume as a fraction of ICV, WMH fraction = white matter hyperintensity volume as a fraction of ICV, SD = Standard deviation, BP = Blood pressure.

*ICV was defined as the sum of whole brain volume and CSF

**Table 2:** Whole brain and cortical GM regions: Mean volume, mean CBF and between-subject standard deviation (SD_B_) for both scanning sessions (r1 and r2) in resting state, within-subject standard deviation (SD_w_), repeatability index (RI), coefficient of variation (CoV) and intraclass correlation (ICC) of CBF, n = 17.

Brain ROI	Volume (ml)	CBF (r1)*	CBF (r2)*	SD_w_ *	RI*	CoV	ICC	P-value†
	mean±SD_B_	mean±SD_B_	mean±SD_B_			(%)		
*Whole brain regions*								
Whole brain parenchyma	1111.1±78.6	38.3±7.8	38.7±7.8	1.12	3.10	2.99	0.98	0.27
Whole brain GM	682.1±42.8	40.6±8.4	41.4±8.7	1.62	4.50	4.10	0.96	0.18
Whole brain normal WM	404.7±46.3	35.2±7.1	35.1±6.3	1.34	3.71	3.93	0.96	0.78
Whole brain total WM	429.1±46.5	34.6±7.2	34.5±6.5	1.34	3.72	4.00	0.96	0.79
Whole brain WMH	24.4±24.5	24.4±4.4	24.4±4.8	2.16	5.98	9.11	0.77	0.97
*Cortical GM regions*								
Orbitofrontal cortex	146.5±11.9	45.2±9.0	45.9±9.7	2.34	6.48	5.29	0.93	0.38
Precentral gyrus	25.0±2.8	39.8±9.1	40.3±10.3	2.55	7.06	6.56	0.93	0.59
Cingulate gyrus	26.0±2.2	52.4±8.5	53.7±9.1	4.34	12.02	8.44	0.74	0.38
Parietal lobe	86.9±7.6	37.4±10.5	37.8±11.1	1.72	4.77	4.71	0.97	0.52
Occipital lobe	85.8±11.0	32.8±10.6	33.7±10.7	2.18	6.04	6.75	0.96	0.24
Medial temporal lobes	21.3±1.8	44.0±5.4	45.1±5.8	2.08	5.77	4.83	0.85	0.14
Lateral temporal lobes	95.7±8.3	41.6±9.0	42.3±9.5	2.20	6.11	5.41	0.94	0.35
Occipitotemporal gyrus	11.5±1.2	37.9±6.8	39.5±8.0	2.14	5.93	5.69	0.91	0.04
Insula	13.7±1.2	43.3±6.4	42.9±6.5	2.53	7.03	6.06	0.84	0.61

*In units of ml/100g/min

†P-value refers to results from the test (paired t-test) of a statistical difference in CBF between the two scans acquired in resting state for every region.

**Table 3:** Subcortical GM regions and WM regions: Mean volume, mean CBF and between-subject standard deviation (SD_B_) for both scanning sessions (r1 and r2) in resting state, within-subject standard deviation (SD_w_), repeatability index (RI), coefficient of variation (CoV) and intraclass correlation (ICC) of CBF, n = 17.

Brain ROI	Volume (ml)	CBF (r1)*	CBF (r2)*	SD_w_ *	RI*	CoV	ICC	P-value†
	mean±SD_B_	mean±SD_B_	mean±SD_B_			(%)		
*Subcortical GM regions*								
Hippocampus	5.5±0.6	47.2±7.9	48.9±8.5	3.86	10.69	8.28	0.76	0.22
Amygdala	4.9±0.5	38.1±6.7	39.8±7.1	4.69	12.98	12.41	0.51	0.31
Thalamus	15.2±1.1	53.6±8.3	55.9±8.1	3.42	9.49	6.45	0.81	0.07
Caudate nucleus	7.2±0.9	46.3±6.5	46.5±7.4	3.33	9.22	7.39	0.76	0.82
Globus pallidus	2.3±0.4	43.0±7.0	43.0±6.1	5.15	14.28	12.36	0.35	0.99
Putamen	9.4±1.1	46.6±7.5	47.8±7.7	3.51	9.72	7.66	0.78	0.33
Nucleus Accumbens	1.0±0.1	59.3±13.0	55.3±11.0	7.91	21.95	14.28	0.50	0.20
*WM regions*								
Frontal lobe	141.2±17.9	35.2±8.4	34.9±7.1	1.75	4.86	5.16	0.95	0.57
Parietal Lobe	75.1±11.2	31.0±7.6	30.4±7.1	1.74	4.81	5.83	0.94	0.37
Temporal Lobe	66.3±8.8	35.5±7.7	34.9±7.0	1.50	4.16	4.39	0.96	0.31
Occipital Lobe	54.6±6.7	28.3±7.9	28.4±7.7	2.34	6.49	8.52	0.91	0.97
External capsule	4.9±0.5	38.0±8.8	37.5±6.0	4.35	12.05	11.86	0.65	0.73
Internal capsule	16.6±2.0	39.4±6.5	39.0±4.9	4.03	11.17	10.62	0.49	0.77

*In units of ml/100g/min

†P-value refers to results from the test (paired t-test) of a statistical difference in CBF between the two scans acquired in resting state for every region.

The CBF (mean±SD) for the whole brain GM was 40.6±8.4 and 41.4±8.7 ml/100g/min for the first and second scan respectively. The corresponding CBF values for normal WM were 35.2±7.1 and 35.1±6.3 ml/100g/min and for total WM 34.6±7.2 and 34.5±6.5 ml/100g/min (Table 2). The GM/WM CBF ratio based on the first scan in rest was 1.2±0.1. The difference in CBF between normal WM and total WM was statistically significant for both scans (p<0.01).

Women had higher CBF values than men for whole brain (42.3±7.4 vs. 35.8±7.2), whole brain GM (45.2±8.1 vs. 38.0±7.7), whole brain normal WM (37.8±6.5 vs. 33.3±6.4) and whole brain total WM (37.5±6.5 vs. 32.5±6.5). This difference was not statistically significant for any of these regions.

**Fig 3:**
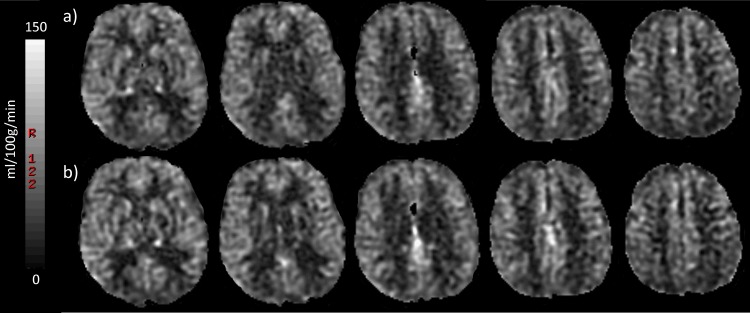
Representative CBF maps of one study participant. Representative CBF maps of an 80 year old male participant with whole brain CBF 47.6 and 49.5 ml/100g/min for the first and second scan at rest respectively. (a) First row corresponds to first scan at rest and (b) second row to rescan at rest.

**Fig 4:**
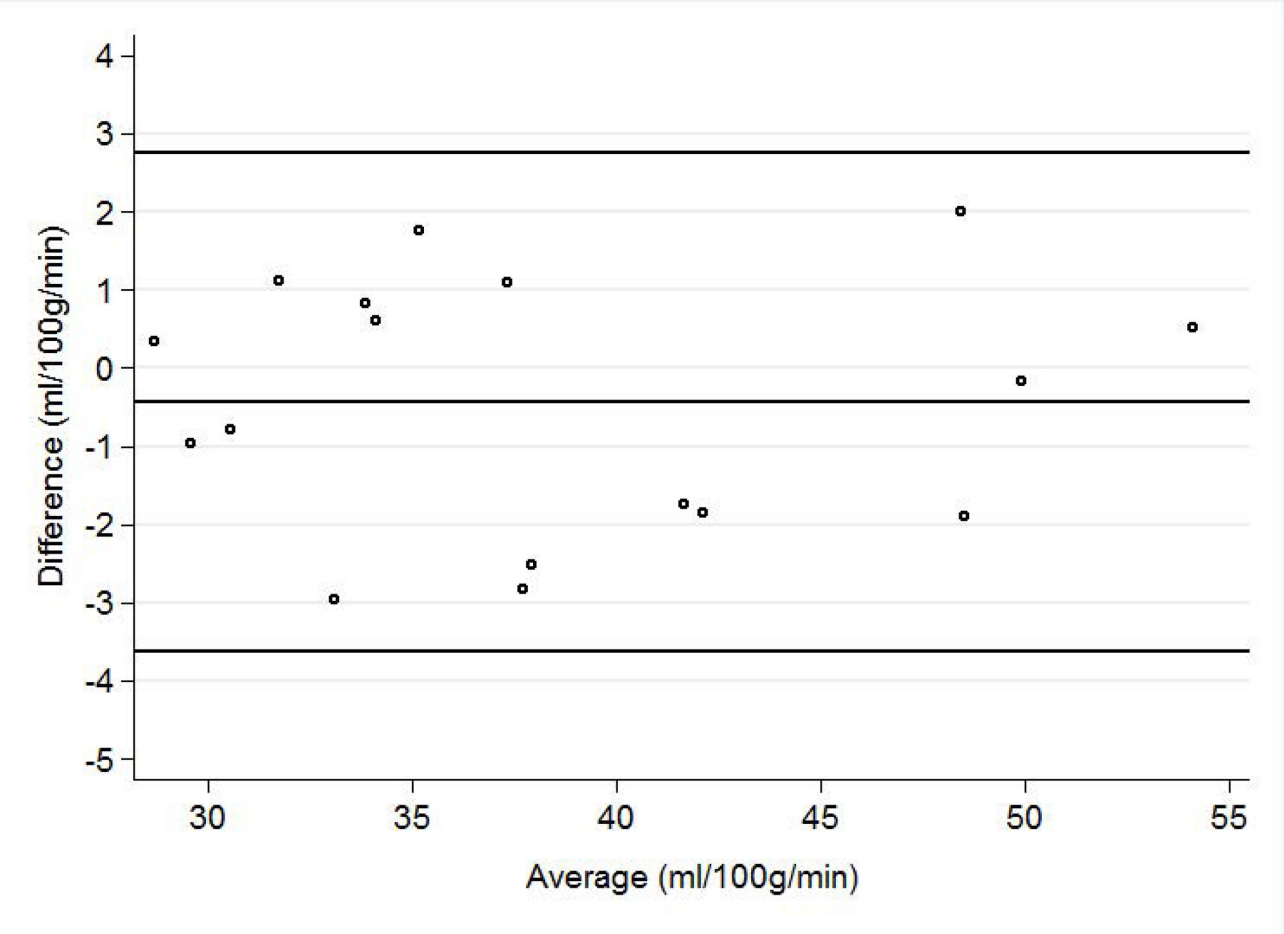
Agreement between scans at rest for whole brain CBF. Bland-Altman plot demonstrating the agreement between the first and second scan at rest for whole brain CBF. Solid mid and upper-lower lines are the mean bias and limits of agreement (1.96 standard deviation), respectively. Y-axis displays mean difference between first and second scan and x-axis the average CBF of the first and second scan.

Results of repeatability (SD_w_, RI, CoV) for whole brain GM were 1.62 ml/100g/min, 4.50 ml/100g/min and 4.10% respectively, for whole brain normal WM 1.34 ml/100g/min, 3.71 ml/100g/min and 3.9% respectively and for whole brain WMH 2.16 ml/100g/min, 5.98 ml/100g/min and 9.11% respectively (representative CBF maps of whole brain CBF for one study participant are shown in Fig 3). Reliability (ICC) for those regions was 0.96, 0.96 and 0.77 respectively (Table 2). A Bland-Altman plot demonstrates the agreement within limits (1.96 standard deviation) between the first and second scan at rest for whole brain CBF in all study subjects (Fig 4).

Repeatability in all other regions ranged from 1.72 to 7.91 ml/100g/min, 3.10 to 21.95 ml/100g/min and 4.71 to 14.28% for SD_w_, RI, CoV respectively. The reliability for these regions varied between 0.35 and 0.97. Variability in CBF was higher in WM regions and subcortical GM compared to cortical GM regions (Tables 2 and 3). Lobar WM reliability was high ranging from 0.91 to 0.96, but lower in the external and internal capsules; 0.65 and 0.49 respectively (Table 3).

### pCASL—Brain activation

**Fig 5:**
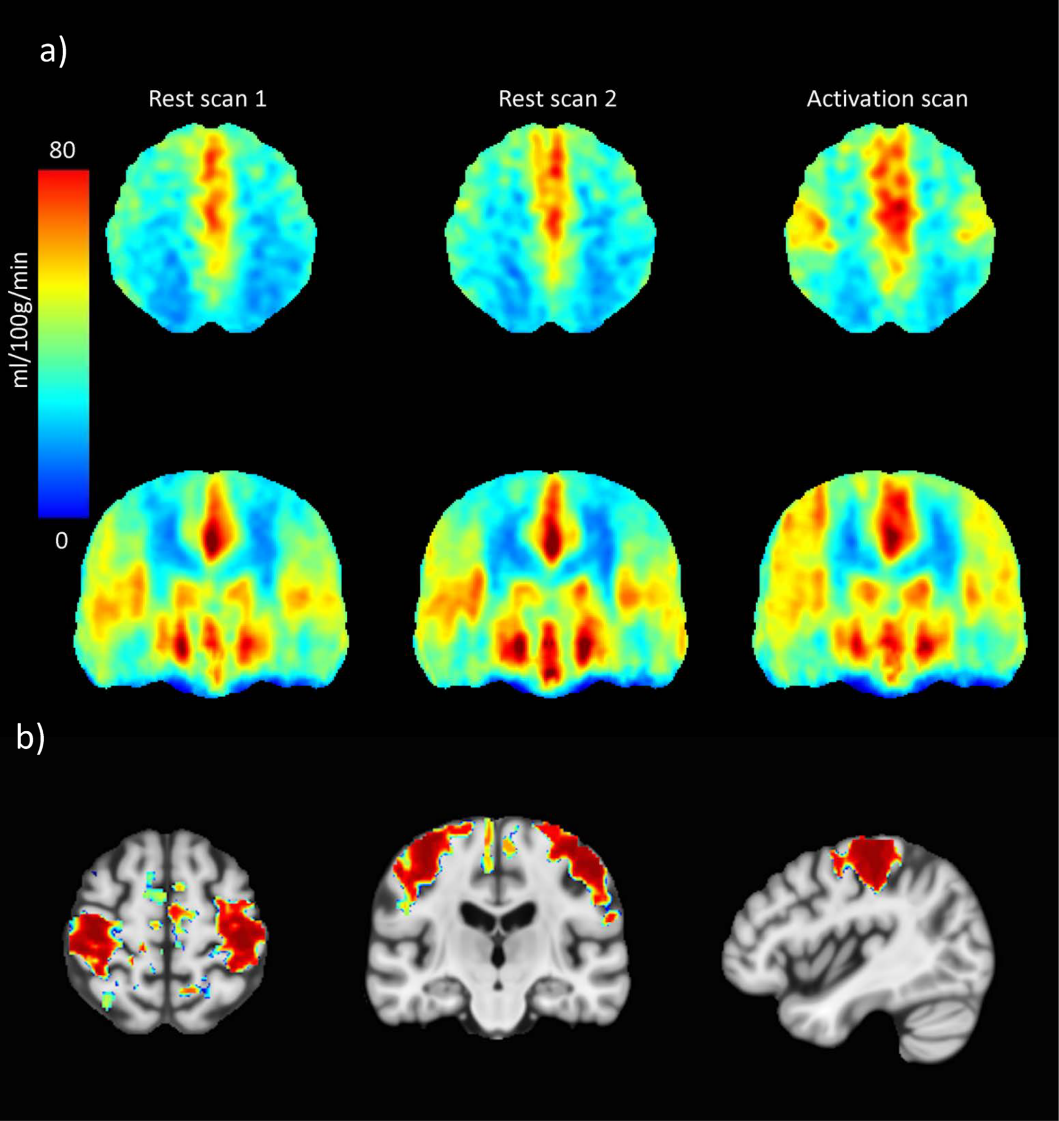
Comparison of CBF values at rest and activation. (a) Average CBF maps of the entire study sample (n = 17) of the first scan at rest (Rest 1), rescan at rest (Rest 2) and at activation by bilateral finger-tapping (activation). (b) Comparison of CBF values at rest and activation by finger-tapping on a voxel level showed significantly higher perfusion during activation in the motor and somatosensory regions at threshold p≤0.01.

Table 4 includes mean pCASL CBF values for scans acquired at rest compared to the activation scans. The mean CBF for whole brain GM was 40.6±8.4 ml/100g/min at rest and 42.6±8.6 ml/100g/min during activation. Of the 17 GM ROIs included in the assessment, six which all were cortical ROIs, showed significantly lower mean CBF at rest compared to activation (p<0.001) (Fig 5A). Comparison of CBF values at rest and activation on a voxel level showed significantly higher perfusion during activation in the motor- and somatosensory regions at threshold p≤0.01 after correcting for multiple comparisons (Fig 5B).

**Table 4:** Mean CBF values at rest (r1) compared to activation (a) by finger tapping in 17 grey matter regions n = 17.

Grey matter ROI	CBF (r1)*	CBF (a)*	P-value†
	mean±SD	mean±SD	
Whole brain	40.6±8.4	42.6±8.6	0.0008
Orbitofrontal cortex	45.2±9.0	46.7±8.8	0.009
Precentral gyrus	39.8±9.1	45.3±11.0	<0.0001
Cingulate gyrus	52.4±8.5	53.2±8.7	<0.0001
Parietal lobe	37.4±10.5	41.4±11.2	<0.0001
Occipital lobe	32.8±10.6	34.8±10.9	0.005
Medial temporal lobes	44.0±5.4	45.3±6.3	0.1
Lateral temporal lobes	41.6±9.0	42.4±9.0	0.2
Hippocampus	47.2±7.9	48.1±7.5	0.5
Amygdala	38.1±6.7	40.4±6.7	0.1
Occipitotemporal gyrus	37.9±6.8	38.9±7.6	0.1
Insula	43.3±6.4	43.8±6.1	0.7
Thalamus	53.6±8.3	55.7±7.2	0.1
Caudate nucleus	46.3±6.5	47.2±6.5	0.5
Globus pallidus	43.0±7.0	45.1±6.7	0.2
Putamen	46.6±7.5	47.1±6.7	0.6
Nucleus Accumbens	59.3±13.0	55.8±10.9	0.1

*In units of ml/100g/min.

†P-value refers to results from the test (paired t-test) of a statistical difference in CBF between the two scans acquired in resting state and during activation for every region.

### Reliability of pCASL compared to PC-MRI

The mean CBF for whole brain measured with PC-MRI was 54.3±10.1 ml/100g/min and with pCASL it was 38.3±7.8 ml/100g/min. The difference was statistically significant (p<0.0001). As with pCASL, women showed on average higher mean whole brain CBF with PC-MRI than men (57.3±11.1 vs. 52.3±9.3, ml/100g/min, p = 0.3). Measurements of CBF using pCASL showed high level of reliability when considering CBF using PC-MRI as a reference standard; the ICC between PC-MRI and pCASL was 0.80.

## Discussion

The aim of this study was to evaluate the feasibility of using perfusion imaging with pCASL on a 1.5-Tesla MRI system in an elderly population. The motivation of the study was the increasing clinical use of ASL and great promises of ASL for the assessment of neurodegenerative disorders as evidenced by fast growing literature [6, 25, 26].

The mean CBF estimates at rest for whole brain GM of this study are consistent with values reported in the PET and ASL MRI literature for healthy elderly individuals [5, 25, 27, 28], while estimates for total WM CBF are in the upper range of other ASL studies [21, 28]. Further, the GM/WM CBF ratio (1.2±0.1) was considerably lower in this study compared to results in many other ASL studies, typically reporting ratios between 2 and 4 [28, 29]. However, these studies used 2D readout sequences in contrast to this study, which used a 3D spiral readout sequence. Three previous studies compared 3D- to 2D readout sequences and reported lower ratio in 3D readout; 1.2 vs 2.1 [30], 1.7 vs 3.9 [31] and 2.2 vs 4.3 [32] due to image blurring [31] and larger extent of spatial smoothing of a spiral 3D readout compared to 2D readout leading to more contamination of the GM signal to WM and vice versa [32]. Another contributor to inaccuracies in WM CBF values in this study may be related to long blood arrival times in WM and shorter T1 of blood at 1.5-Tesla compared to scanners with higher field strength [9, 33, 34]. Investigators and users of ASL should therefore interpret perfusion in WM with caution. Increasing age additionally contributes to lower GM/WM CBF ratio [28].

The mean CBF for total WM were significantly lower than mean CBF in normal WM. Hypoperfusion in areas of WMH is consistent with findings in two other studies [35, 36] and show that differences in WMH volume should be accounted for.

Women had on average higher CBF values than men for whole brain regions although this difference was not statistically significant. Higher CBF in women compared to men is in accordance with other ASL perfusion studies [3, 11, 27] and may be explained by lower hematocrit levels in women and therefore longer T1-relaxation time of blood [8]. The quantification of CBF in this study assumed the same T1 of blood in men and women in accordance to the recent paper by Alsop et al on the recommended implementation of ASL perfusion for clinical applications [6]. In the study by Zhang et al [8], the T1 of blood at 1.5-Tesla was measured as 1430 msec in men and 1530 msec in women. The CBF in whole brain GM in this study was found to be 16% higher in women compared to men on average. After correcting for blood T1 differences in men and women based on these T1 values using the CBF quantification method recommended by Alsop et al [6] for pCASL, this difference would be reduced to 5%. That together with the fact that the CBF values with PC-MRI were also higher in women compared to men suggests that the longer T1 of blood in women explains most, but not all of the observed sex difference.

### Repeatability and reliability

The estimates of inter-session reproducibility and reliability in this study do not suggest increased variability in perfusion of healthy elderly subjects when compared to other ASL studies of younger subjects. In a study by Wang et al [37], reproducibility of CBF by pulsed ASL at 3 Tesla was assessed in multiple brain regions in 10 healthy young adults and showed inter-session variability ranging from 2.0 to 8.8 (SDw) and ICC ranging from 0.68 to 0.94. The SDw and ICC for whole brain cortical GM was 3.3 and 0.90 respectively. In another study using pCASL at 3-Tesla [28], the inter-session ICC in 8 young adults ranged from 0.82 to 0.98 for whole brain WM, lobar GM and posterior cingulate gyrus. These results are comparable to the results from this study. Less evidence exists in the current literature on ASL reproducibility in older individuals and results are inconsistent. The ICC in the same study as referenced to above [28], but in an older group of subjects (n = 14, age range 50–73 years) ranged from 0.80 to 0.96 for the same brain regions. In a recent study addressing inter-session reproducibility at 3-Tesla in older cognitively impaired subjects and normal controls, the ICCs of global brain perfusion were 0.70 and 0.41 for a 3D- and a 2D based pCASL sequences respectively [38] which is considerably lower compared to this study.

The reproducibility and reliability in the present study was high for most regions. The between-subject SD was up to seven times larger than SD_W_ for all 27 regions, there was no statistically significant difference between the two scans at rest for 26 of the 27 regions and the ICC reflected moderate to high reliability for 24 out of the 27 regions (Tables 2 and 3). Smaller ROI sizes contribute to increased within-subject variability as evidenced by inverse correlation between regional volume and SD_W_, consistent with the results of Asllani et al [39].

Smaller SD_W_ compared to SD_B_ is in agreement with previous ASL perfusion regional reproducibility studies [21, 37]. This together with moderate to high ICCs for most regions supports that within-subject effects are a much smaller contributor to variability in perfusion measurements than other sources of variability between subjects, not only in young adults [37] but also in old individuals. Other sources of variability have been suggested to include variation in neuronal density, individual differences in underlying physiological fluctuations and T1 relaxation time of blood [2, 3, 40].

### Brain activation

Another part of this study included brain activation measurements to assess the ability of ASL in detecting flow differences. The results showed clear flow differences between rest and activation in the expected brain regions. The anatomical location of the hand motor area (precentral gyrus) and the sensory receptive area for the sense of touch (postcentral gyrus) have been well established in the literature [41]. The mean CBF difference between the rest and activation scan was statistically significant for 6 out of the 17 GM regions, all but one (occipital lobe) adjacent to or overlapping with the precentral or postcentral gyrus (Table 3). Further, the comparison on a voxel level showed significantly higher perfusion during activation in the motor- and somatosensory regions (Fig 5B).

### Reliability of pCASL compared to PC-MRI

The reliability of pCASL was also tested by comparing it to PC-MRI as a reference standard. Flow quantification with PC-MRI is a well-established, robust method [42] that has been used in at least one previous study [4] as reference standard in the assessment of ASL perfusion reliability in children and showed a reliability of 0.65 (ICC). In the current study the ICC of pCASL compared to PC-MRI was higher (0.80).

### Study limitations

This study has several limitations. Small sample size may explain lack of statistical significance in perfusion sex differences. The non-triggered PC-MRI scan used as a reference standard may contain noise and ghosting due to cardiac pulsation and physiologic fluctuations resulting in inaccuracies in the flow quantification. Further, averaging flow information over the cardiac cycle compared to trigger the scan to the cardiac cycle has been shown to give rise to a loss of information in the periphery of the vessels, resulting in artificially high flow values [19]. The assumed T1-relaxation time of blood in this study (1400 msec) was somewhat low compared to measured T1 of blood in the study by Zhang et al (1430 msec in men and 1530 in women) [8].

## Conclusions

In conclusion, the overall results of this study demonstrate moderate to high level of repeatability and reliability for majority of regions, comparable to what has been reported in younger populations with 3.0 Tesla MRI systems. The performance of pCASL on a 1.5-Tesla system in this study shows that perfusion measurements in brain regions of very old individuals are feasible.
